# Effects of Remotely Delivered Yoga Interventions on Quality of Life and Symptom Burden in Women with Breast Cancer: A Systematic Review and Meta-Analysis

**DOI:** 10.3390/healthcare14183040

**Published:** 2026-09-16

**Authors:** Federica Valeriani, Francesca Gallè, Giulia Scalese, Romy Angela Valia, Marco Ferrante, Daniela Marotta, Andrea Botticelli, Monica Verrico, Carmela Protano

**Affiliations:** 1 Department of Movement, Human and Health Sciences, University of Rome Foro Italico, 00135 Rome, Italy; federica.valeriani@uniroma4.it; 2 Department of Medical, Movement and Wellbeing Sciences, University of Naples Parthenope, 80133 Naples, Italy; 3 Department of Public Health and Infectious Diseases, Sapienza University of Rome, 00185 Rome, Italy; giulia.scalese@uniroma1.it (G.S.); romyangela.valia@uniroma1.it (R.A.V.); marco.ferrante@uniroma1.it (M.F.); monica.verrico@uniroma1.it (M.V.);; 4 Department of Prevention, Local Health Authority Rome 2, 00157 Rome, Italy; daniela.marotta@aslroma2.it; 5 Department of Radiological, Oncological and Pathological Science, Sapienza University of Rome, 00161 Rome, Italy; andrea.botticelli@uniroma1.it

**Keywords:** breast cancer survivors, yoga, digital health, telehealth, remote intervention, quality of life, home workout, supportive care

## Abstract

**Background/Objectives:** Breast cancer (BC) survivors frequently experience persistent psychological and physical symptoms and reduced health-related quality of life (HRQoL). Yoga has emerged as a promising supportive intervention in this context; however, evidence regarding remotely delivered yoga programs remains limited. This systematic review aimed primarily to evaluate the effects of remotely delivered yoga interventions on HRQoL among women with BC. Secondary outcomes included psychological and physical symptoms, as well as feasibility, adherence, and safety. **Methods:** Randomized controlled trials (RCTs) and non-randomized studies assessing remote, home-based, or hybrid yoga interventions delivered through digital platforms, videos, or remote support were searched in PubMed, Scopus, Web of Science, and the Cochrane Library (PROSPERO registration number: CRD420261362619). Methodological quality was assessed using the Joanna Briggs Institute (JBI) critical appraisal tools. A random-effects meta-analysis was performed for HRQoL outcomes from independent RCT populations, using standardized mean differences (Hedges’ g). **Results:** Twenty-six publications representing 19 independent study cohorts were included. Eighteen publications reported randomized controlled trials and eight reported non-randomized studies. In the meta-analysis, the 9 RCTs that reported effects on HRQoL showed a moderate improvement in this outcome (Hedges’ g = 0.56, 95% CI 0.17–0.96), with substantial between-study heterogeneity (I^2^ = 72.4%). Secondary psychological and physical outcomes showed potential benefits, but findings varied across studies. Feasibility and adherence were generally favorable, while no serious yoga-related adverse events were reported among studies assessing safety. **Conclusions:** Remote and home-based yoga interventions may improve HRQoL in women with BC; however, the consistency and generalizability of this effect are limited by substantial between-study heterogeneity and variability in intervention delivery. Further standardized and adequately powered RCTs are needed to confirm clinical effectiveness and inform long-term implementation in breast cancer care.

## 1. Introduction

Despite improvements in early detection and treatment, Breast Cancer (BC) remains a major global health challenge and a leading cause of cancer burden among women. BC is the most frequently diagnosed malignancy among women worldwide and is responsible for over a quarter of all female cancer cases and still represents a predominant global health challenge [1,2]. According to the Global Cancer Statistics, in 2022 BC accounted for approximately 2.3 million new cases worldwide, representing 11.6% of all cancer diagnoses, and caused nearly 670,000 deaths [2]. Advances in neoadjuvant and adjuvant systemic therapies, targeted treatments, and surgical management have substantially improved survival outcomes and reduced recurrence rates among patients with BC [3]. However, many survivors continue to experience a broad range of persistent treatment-related adverse effects and sequelae. Physical and psychological symptoms such as cancer-related fatigue (CRF), sleep disturbances, anxiety, depression, cognitive impairment, sexual dysfunction, and body image concerns frequently persist long after completion of treatment, significantly compromising quality of life (QoL) and overall well-being. These unmet supportive care needs have prompted growing interest in evidence-based integrative interventions aimed at improving survivorship outcomes [4].

Among these interventions, yoga has emerged as one of the most widely studied mind–body therapies in oncology [5,6,7]. Yoga combines physical postures (asanas), breathing techniques (pranayama), and meditation practices (dhyana), offering a multidimensional approach that addresses both physical and psychological aspects of recovery [8]. Increasing evidence suggests that yoga may modulate stress-related physiological pathways, improve autonomic regulation, and reduce symptom burden in cancer populations. Moreover, yoga has been associated with improvements in psychological distress, anxiety, depressive symptoms, sleep quality, and health-related quality of life (HRQoL), while also showing potential benefits in reducing systemic inflammatory activity [9,10,11,12,13]. In BC survivors, randomized controlled trials and meta-analyses have demonstrated that yoga may effectively alleviate CRF, improve mood and emotional well-being, reduce pain and sleep disturbances, and enhance overall HRQoL during and after treatment [14,15]. Consequently, yoga is increasingly recommended as a supportive care strategy within integrative oncology programs for patients with BC [16].

Traditionally, yoga interventions for individuals with cancer have been delivered through face-to-face sessions, allowing direct supervision by trained instructors, real-time feedback, and individualized adaptation of postures to ensure safety and optimize therapeutic benefits. However, participation in in-person activities may be hindered by several barriers, including transportation difficulties, financial constraints, physical limitations linked to oncological treatment, and immunocompromised conditions that may restrict attendance in conventional yoga sessions [17,18,19].

The rapid evolution of digital health technologies—drastically accelerated by the necessity of remote solutions during the COVID-19 pandemic—has fundamentally transformed this paradigm [20]. Today, exercise and supportive care are widely accessible through interactive live-streamed classes, web-based platforms, mobile applications and asynchronous, pre-recorded video modules/audiocassettes. These remote delivery modalities offer unprecedented adaptability, flexibility, and convenience, allowing patients to practice in their own space within the comfort and safety of their homes.

Nevertheless, remote yoga interventions are inherently heterogeneous. Synchronous formats preserve real-time interaction and instructor supervision, whereas asynchronous modalities offer greater flexibility and scalability but provide limited opportunities for personalized feedback and monitoring. Such differences may influence adherence, engagement, safety, and ultimately clinical effectiveness. Consequently, the comparative effectiveness of different remote yoga delivery modalities remains uncertain.

Although an increasing number of randomized controlled trials (RCTs) have investigated yoga-based interventions in women with BC, the relative efficacy of distinct delivery formats has not been comprehensively established [21]. Given the growing global burden of BC and the sustained integration of telehealth into contemporary oncology care, a rigorous synthesis of the available evidence is warranted.

The present systematic review and meta-analysis aim to evaluate the effectiveness of remotely delivered yoga interventions in women with BC, with HRQoL as the primary outcome. Secondary outcomes include psychological and physical symptoms, including CRF and sleep quality, as well as feasibility, adherence, and safety. By synthesizing evidence from randomized and non-randomized studies, this study seeks to inform evidence-based supportive care and clinical decision-making in oncology.

## 2. Materials and Methods

The study was conducted in accordance with the PRISMA 2020 guidelines for systematic reviews (Supplementary Figures S2 and S3) [22]. The protocol was registered in the PROSPERO database (registration number: CRD420261362619) and is available at: https://www.crd.york.ac.uk/PROSPERO/view/CRD420261362619 (accessed on 25 July 2026). Two independent reviewers (G.S. and R.A.V.) performed study selection, data extraction, and quality assessment. Any discrepancies were resolved through discussion; if consensus could not be reached, a third reviewer (D.M.) provided the final decision.

### 2.1. Literature Search Strategy

A systematic literature search was conducted on 26 March 2026 in the following databases: MEDLINE (via PubMed), Scopus, Web of Science, and the Cochrane Library, from database inception to the search date. The search strategy combined controlled vocabulary (MeSH terms) and free-text terms, structured around three main concepts: (1) breast cancer, (2) yoga interventions, and (3) remote/digital delivery modalities. For the population, terms included breast cancer, breast carcinoma, neoplasm, and related variants, together with MeSH terms such as “Breast Neoplasms” and “Neoplasms”. For the intervention, terms included yoga, asana, pranayama, specific styles (e.g., Hatha, Iyengar, Vinyasa, Kundalini), and the MeSH term “Yoga”. For delivery modalities, terms included descriptors of remote and digital interventions such as online, web-based, internet, tele-yoga, telehealth, e-health, mobile app, videoconference, video-based, and home-based, as well as supervision-related terms (e.g., guided, instructor-led, supervised, self-guided, unsupervised). Boolean operators (AND/OR) were used to combine terms within and across domains. The PubMed strategy was developed first and subsequently adapted for the other databases according to their specific syntax. The full search strings for all four databases are provided in the Supplementary Material (Supplementary Table S1).

### 2.2. Eligibility Criteria

Eligible studies included randomized controlled trials (RCTs) and non-randomized interventional studies enrolling adult women (≥18 years) diagnosed with BC.

Studies were included only when the intervention involved yoga delivered through remote or home-based modalities, including live-streamed, videoconference-based, web-based, mobile application-based, or pre-recorded formats. A minimum threshold of >50% of the total intervention exposure (based on number of sessions or total prescribed practice time, when reported) had to be delivered remotely for a study to be considered eligible. Hybrid interventions in which the majority of sessions were delivered face-to-face were excluded.

Eligible interventions were required to include structured physical yoga practice involving movement-based components (asanas/postures and/or yoga flows). Interventions limited to pranayama or breathing-based practices were also excluded because the objective of this review was to evaluate yoga as a multidimensional body intervention incorporating a measurable physical activity component. Furthermore, interventions consisting exclusively of mindfulness, meditation, or relaxation techniques without physical yoga postures were excluded as well. Similarly, interventions described as “laughter yoga” or other yoga-derived practices without structured physical exercise components were excluded.

On the contrary, yoga interventions in which mindfulness or meditation practices were integrated as complementary components within a structured yoga program (e.g., Hatha yoga, Iyengar yoga, or therapeutic yoga) were considered eligible, provided that physical practice remained the core component of the intervention.

Studies enrolling participants with different cancer diagnoses were eligible only when breast cancer-specific data were reported separately or could be obtained from the published results. When mixed samples were reported, studies were included in the quantitative synthesis only if outcome data for BC participants were available separately. Otherwise, they were excluded from meta-analysis.

Specifically, only outcomes referring exclusively to female BC participants were extracted whenever subgroup-level data were available; otherwise, the study was excluded from quantitative synthesis.

### 2.3. Outcomes of Interest and Quality Assessment

The primary outcome was HRQoL. Secondary outcomes included sleep quality, CRF, anxiety, depression, pain, overall symptom burden, feasibility, intervention adherence, and safety. Methodological quality was assessed using the Joanna Briggs Institute (JBI) critical appraisal tools [23,24].

### 2.4. Data Extraction and Outcome Selection

Data extraction was independently performed using a standardized form following recommendations from the Cochrane Handbook for Systematic Reviews of Interventions. For the primary meta-analysis, only studies reporting HRQoL outcomes and sufficient quantitative data to calculate standardized mean differences (sample size, post-intervention or change mean values, and corresponding standard deviations for both intervention and control groups) were included. When multiple HRQoL measures were reported within the same study, only one measure was selected according to a predefined hierarchy to avoid double-counting participants. Preference was given to validated HRQoL instruments, including Functional Assessment of Cancer Therapy-General (FACT-G), Functional Assessment of Cancer Therapy-Breast (FACT-B and FACT-B+4), EORTC Quality of Life Questionnaire Core 30 (QLQ-C30), Lymphoedema Quality of Life Questionnaire (LYMQOL), Functional Living Index for Cancer (FLIC), and Lymphedema Life Impact Scale (LLIS) [25]. Studies reporting only psychological outcomes, such as depression, anxiety, stress, or self-esteem, were not included in the primary HRQoL meta-analysis; these outcomes were considered separately as secondary outcomes in the narrative synthesis, consistent with previous reviews distinguishing quality-of-life outcomes from psychological health outcomes in cancer populations [26,27].

For the primary HRQoL meta-analysis, post-intervention scores were extracted; when multiple follow-up assessments were available, the first assessment immediately after completion of the intervention was selected for the primary analysis. For each HRQoL outcome, sample size, mean values, and standard deviations (SDs) were extracted for both intervention and control groups. When studies reported standard errors (SEs), SDs were calculated using the formula SD = SE × √n, as recommended by the Cochrane Handbook [28]. In studies where only change-from-baseline values were available, such as Stan et al. [29], effect sizes were calculated using mean changes and corresponding SDs.

To enable comparison across different HRQoL instruments, outcome directions were harmonized before analysis. For scales in which higher scores represented better health status or well-being (e.g., FACT-G, FACT-B, FACT-B+4, EORTC QLQ-C30, and FLIC), data were used as reported. For scales in which lower scores reflected better outcomes (e.g., LYMQOL and LLIS), the direction of the effect size was reversed so that positive values consistently reflected a beneficial effect of yoga. This procedure is recommended when combining patient-reported outcomes measured on scales with opposite directions [28].

Since different HRQoL instruments were used across studies, pooled effects were estimated using standardized mean differences (Hedges’ g) and corresponding 95% confidence intervals. Hedges’ g was preferred because it corrects for small-sample bias and is recommended when combining continuous outcomes assessed using different scales [28,30]. For exploratory moderator analyses, study and intervention characteristics were categorized as follows. Countries were classified according to the World Health Organization (WHO) regional framework (Region of the Americas, European Region, Eastern Mediterranean Region, South-East Asia Region, and Western Pacific Region). Clinical setting was categorized as active treatment, survivorship, or breast cancer-related lymphedema. Yoga interventions were classified as Traditional Yoga, Therapeutic Yoga, General Yoga, or Yoga-Based Relaxation according to their primary theoretical framework and clinical adaptation. Delivery mode was categorized as remote/home-based (supervised via live streaming or unsupervised) or hybrid (combining remote/home-based delivery with in-person sessions). Intervention duration was categorized as short-term (≤8 weeks), medium-term (>8–16 weeks), or long-term (>16 weeks) [31]. Given the limited number of independent studies and the small number of studies within several moderator categories, all moderator analyses were considered exploratory.

To avoid double-counting participants, only one effect estimate from each independent study population was included in the primary meta-analysis when multiple publications originated from the same or overlapping cohort. Studies that did not provide sufficient descriptive statistics to calculate standardized mean differences or reported only model-based estimates (e.g., regression coefficients, adjusted mean differences, or standardized effect sizes) were excluded from the quantitative synthesis but retained in the qualitative review.

Since lower LLIS values indicate better functioning and lower lymphedema impact, contrary to the other scores, the direction of the standardized mean difference was reversed in the studies where the LLIS score was used, so that positive values consistently indicated a beneficial effect of yoga.

### 2.5. Statistical Analysis

Random-effects meta-analyses were performed to estimate pooled standardized mean differences (Hedges’ g) and corresponding 95% confidence intervals. Between-study variance (τ^2^) was estimated using the Hedges estimator. Confidence intervals and statistical tests for the pooled effect were calculated using the Knapp–Hartung adjustment. Statistical heterogeneity was assessed using Cochran’s Q statistic, τ^2^, and the I^2^ statistic. A 95% prediction interval was calculated to estimate the range within which the true effect of a future comparable study would be expected to lie. Potential outlying and influential studies were evaluated using studentized residuals and Cook’s distances. No statistically significant funnel-plot asymmetry was detected according to Begg and Mazumdar’s rank correlation test (τ = −0.222, *p* = 0.477) or Egger’s regression test (intercept = −1.228, *p* = 0.259). The trim-and-fill procedure imputed one potentially missing study. However, given the small number of independent studies (k = 9) and the substantial between-study heterogeneity, these analyses were considered exploratory. The absence of statistically significant funnel-plot asymmetry should therefore not be interpreted as evidence of absence of publication bias or small-study effects. Exploratory univariate meta-regression analyses were performed to investigate whether publication year and selected study characteristics (WHO region, clinical setting, yoga type, delivery mode, and intervention duration) were associated with variation in effect size. Given the limited number of independent studies included in the primary meta-analysis and the small number of studies within several moderator categories, these analyses were considered exploratory and interpreted with caution. Non-significant associations were not interpreted as evidence of the absence of moderator effects.

## 3. Results

### 3.1. Study Selection

**Figure 1:**
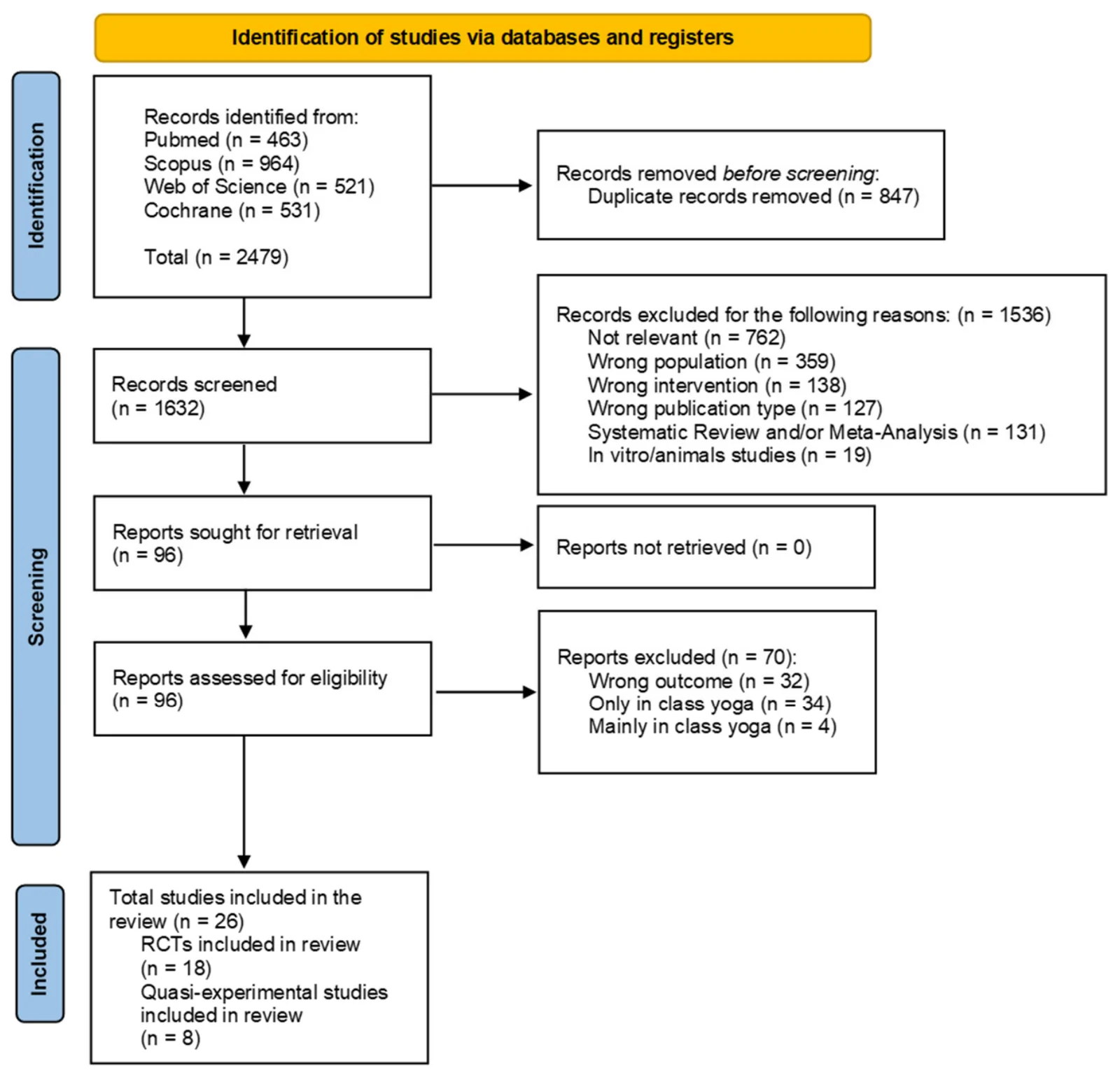
PRISMA 2020 flow diagram illustrating the study selection process for the systematic review. Adapted from the PRISMA 2020 Statement [22].

The systematic search identified a total of 2479 records across PubMed, Scopus, Web of Science, and the Cochrane Library. After removal of 847 duplicates, 1632 records underwent title and abstract screening, resulting in the exclusion of 1536 records that did not meet the eligibility criteria. We assessed for eligibility the full texts of the remaining 96 articles, of which 70 were excluded, primarily because they evaluated predominantly in-person yoga interventions, did not report eligible outcomes, or did not meet the predefined eligibility criteria. Ultimately, 26 publications including 18 RCTs and 8 quasi-experimental studies and representing 19 independent cohort studies fulfilled the inclusion criteria (Figure 1).

### 3.2. Characteristics of Included Studies

Table 1 reports the characteristics of the studies included in the review. Several publications reported secondary analyses, follow-up assessments, or additional outcomes derived from previously published cohorts. To avoid double-counting participants, publications originating from the same study population were considered as a single independent study when summarizing study characteristics. Specifically, the reports by Littman et al. [32] and Cadmus-Bertram et al. [33], Lanctôt et al. [34] and Anestin et al. [35], Prakash et al. 2017 and 2020 [36,37], Raghavendra et al. [38] and Rao et al. [39], together with the three publications by Kovačič and colleagues of 2011, 2011 and 2013 [40,41,42], were treated as single study cohorts. In contrast, although Winters et al. [43] and Flanagan et al. [44] implemented highly comparable interventions, participant overlap could not be confirmed and the studies were therefore considered independent.

Overall, the 26 included publications corresponded to 19 independent study cohorts, enrolling a total of 1109 unique women with BC. Studies were published between 2007 and 2026, with sample sizes ranging from 14 to 173 participants. Participants were recruited across different stages of the BC continuum, including women undergoing active treatment [36,38,45], BC survivors after completion of primary treatment [29,32,46,47], and patients with breast cancer-related lymphedema [48,49,50]. Mean participant age generally ranged from the mid-forties to the early sixties, although individual ages spanned approximately 28 to 80 years.

Considerable variability was observed in the characteristics of remotely delivered yoga interventions. Nine studies evaluated exclusively remote yoga programmes [29,43,44,46,47,51,52,53,54], whereas the remaining studies adopted hybrid models combining supervised face-to-face sessions with prescribed home practice. Synchronous interventions enabled real-time interaction with certified instructors, whereas asynchronous programmes were primarily based on self-directed practice using pre-recorded video or audio materials. Earlier interventions primarily relied on DVDs, printed manuals, booklets, or audio recordings, while more recent studies increasingly incorporated digital platforms, videoconferencing systems, mobile applications, and web-based programmes. Intervention duration ranged from 4 to 26 weeks, with individual sessions lasting between 15 and 120 min. Prescribed practice frequency ranged from one supervised session per week supplemented by home practice to daily practice. Outcome assessments were performed immediately after completion of the intervention in all studies, with several investigations also including longer-term follow-up assessments. Though different yoga styles were employed across the studies, all of them adopted protocols of structured physical yoga practice involving movement-based components.

**Table 1:** Characteristics of the selected studies.

Author (Year), Country, Ref. n.	Study Design	Initial Sample Size	Final Number of Participants	Exercise Duration and Frequency	Delivery Tool
Đorđević et al. (2024), Germany [51]	Randomized controlled trial	173	117	45 min online sessions, twice a week for 6 weeks	Video files on website
Stan et al. (2016), USA [29]	Randomized controlled trial	34	34	20 min home-based sessions, 3–5 times per week for 12 weeks	DVD
Loudon et al. (2014), Australia & New Zealand [48]	Randomized controlled trial	28	19	A weekly 90 min teacher-led class and 40 min home-based daily sessions, for 8 weeks	Class + DVD
Loudon et al. (2016), Australia & New Zealand [49]	Randomized controlled trial				
Prakash et al. (2017), India [36]	Randomized controlled trial	100	100	2 h in-person demonstration and 14 home-based sessions a week, twice a day, for 18 weeks	In-person demonstration + booklet
Prakash et al. (2020), India [37]					
Raghavendra et al. (2007), India [38]	Randomized controlled trial	98	62	Yoga counselling and daily home-based 60 min sessions for 4–8 CT cycles, supervised once every 10 days at home	In person demonstration + audio/video files
Rao et al. (2017), India [39]			69		
Kovačič et al. (2011), Slovenia [40]	Randomized controlled trial	32	32	45 min daily sessions for 4 weeks: 1 supervised in a physiotherapy room, 3 unsupervised at home	In-person class + audio recording files
Kovačič et al. (2011), Slovenia [41]	Randomized controlled trial				
Kovačič et al. (2013), Slovenia [42]	Randomized controlled trial				
Littman et al. (2012), USA [32]	Randomized controlled trial	63	27	2–3 times per week facility- based 75 min classes + 2–4 home-based 20–30 min sessions per week, for 24 weeks	In-person class + DVD/audio/video files
Lanctôt et al. (2015), Canada [34]	Randomized controlled trial	101	92	90 min weekly session + 20–40 min home-based unsupervised daily sessions, for 8 weeks	In-person class + DVD
Anestin et al. (2017), Canada [35]	Randomized controlled trial	84	38 (19 + 19)		
Qi et al. (2026), China [45]	Randomized controlled trial	30	28	3 weekly 60 min facility- or home-based supervised sessions, for 8 weeks	In-person classes + Videos, Tencent Meeting software, booklet, telephone
Harder et al. (2015), UK [46]	Randomized controlled trial	92	78	One in-person meeting and 1 home-based unsupervised weekly 60 min session, for 10 weeks	DVD
Movafegh et al. (2026), Iran [50]	Randomized controlled trial	112	96	2 supervised in-person and home-based weekly 60 min sessions, for 8 weeks	In-person class + Instagram
Ferrigno Guajardo et al. (2025), Mexico [47]	Randomized controlled trial	68	29	1 weekly supervised online 120 min session + unsupervised 20–30 min daily home-based sessions, for 8 weeks	Videoconference class + audio recordings
Zok (2023), Poland [52]	Pre-post intervention single-group study	41	13	1 weekly 75 min online supervised home-based sessions, for 12 weeks	Virtually supervised Zoom class
Winters (2020), USA [43]	Prospective intervention single-group study	14	11	2 weekly home-based 17 min sessions, for 4 weeks	Web-based video files
Komatsu et al. (2016), Japan [55]	Pre-post intervention single-group study	21	18	One 90 min in-person class and 1–3 weekly 15 min home-based unsupervised sessions	Class + DVD + booklet
Lengacher et al. (2017), USA [53]	Pre-post intervention single-group study	15	13	Weekly 120 min sessions, for 6 weeks	Audio/video files for iPad
Faravel et al. (2021), France [56]	Pre-post intervention single-group study	24	22	Period 1: 1 weekly 90 min supervised session + 15 min daily at-home sessions for 6 weeks. Period 2: 15 min daily at-home sessions, for 6 weeks	Class + booklet + audio files
Cadmus-Bertram et al. (2013), USA [33]	Post-intervention study on the intervention group from an RCT	32	32	1–2 weekly 75 min in-person class + 4–3 weekly 15–30 min sessions, for 26 weeks	Class + DVD + booklet
Flanagan et al. (2021), USA [44]	Pre-post intervention single-group study	43	35	7 weekly 20 min home-based sessions, for 4 weeks	Video files
Neville A.R. et al. (2023), Canada [54]	Pre-post intervention single-group study	20	18	2 weekly 60 min online supervised sessions, for 8 weeks	Zoom class

Comparator groups in RCTs included usual care, wait-list controls, supportive counselling, physiotherapy or rehabilitation programs, strengthening exercise, and standard self-care for breast cancer-related lymphedema, whereas all quasi-experimental studies used single-arm pre-post designs.

The main outcomes of the selected studies, with related significance levels, are reported in Table 2. The outcome changes from randomized and non-randomized studies are visually distinguished.

HRQoL was the primary outcome and was investigated in 13 publications. Secondary outcomes included feasibility and adherence, which were analyzed in 15 publications, followed by anxiety (10 publications), CRF and safety (9 publications), depression (8 studies), sleep quality (5 publications) and perceived stress (4 publications). Less frequently assessed outcomes included cognitive function, pain, self-esteem, mobility, treatment-related side effects, lymphedema-related outcomes, and fear of cancer recurrence (Table S2).

### 3.3. Methodological Quality Assessment

Among the 18 RTCs, most studies fulfilled the JBI criteria for randomization, allocation procedures, baseline similarity between groups, outcome measurement, follow-up, and statistical analysis. The main limitations identified across studies concerned the lack of blinding of participants and intervention providers, which was expected given the nature of yoga interventions, and the incomplete reporting of outcome assessor blinding. One trial showed concerns regarding the randomization process [34].

The eight quasi-experimental studies generally showed adequate methodological quality, with appropriate temporal assessment, reliable outcome measurement, and suitable statistical analyses.

However, methodological limitations such as baseline imbalance, unclear randomization, no blinded outcome assessment, incomplete reporting and the lack of control groups were found in several studies. The detailed results of the JBI critical appraisal are presented in Supplementary Tables S3 and S4.

### 3.4. Health-Related Quality of Life

HRQoL was the most frequently evaluated clinical outcome. Overall, the qualitative evidence indicated a general trend towards improved HRQoL following remotely delivered yoga interventions across different stages of the BC continuum. Improvements were reported both in women undergoing active cancer treatment and in BC survivors following completion of primary therapy, although the magnitude of benefit varied across studies according to participant characteristics, intervention protocols, and outcome measures. Findings were less consistent among studies of women with breast cancer-related lymphedema, where improvements assessed with disease-specific QoL instruments were smaller or not statistically significant.

**Figure 2:**
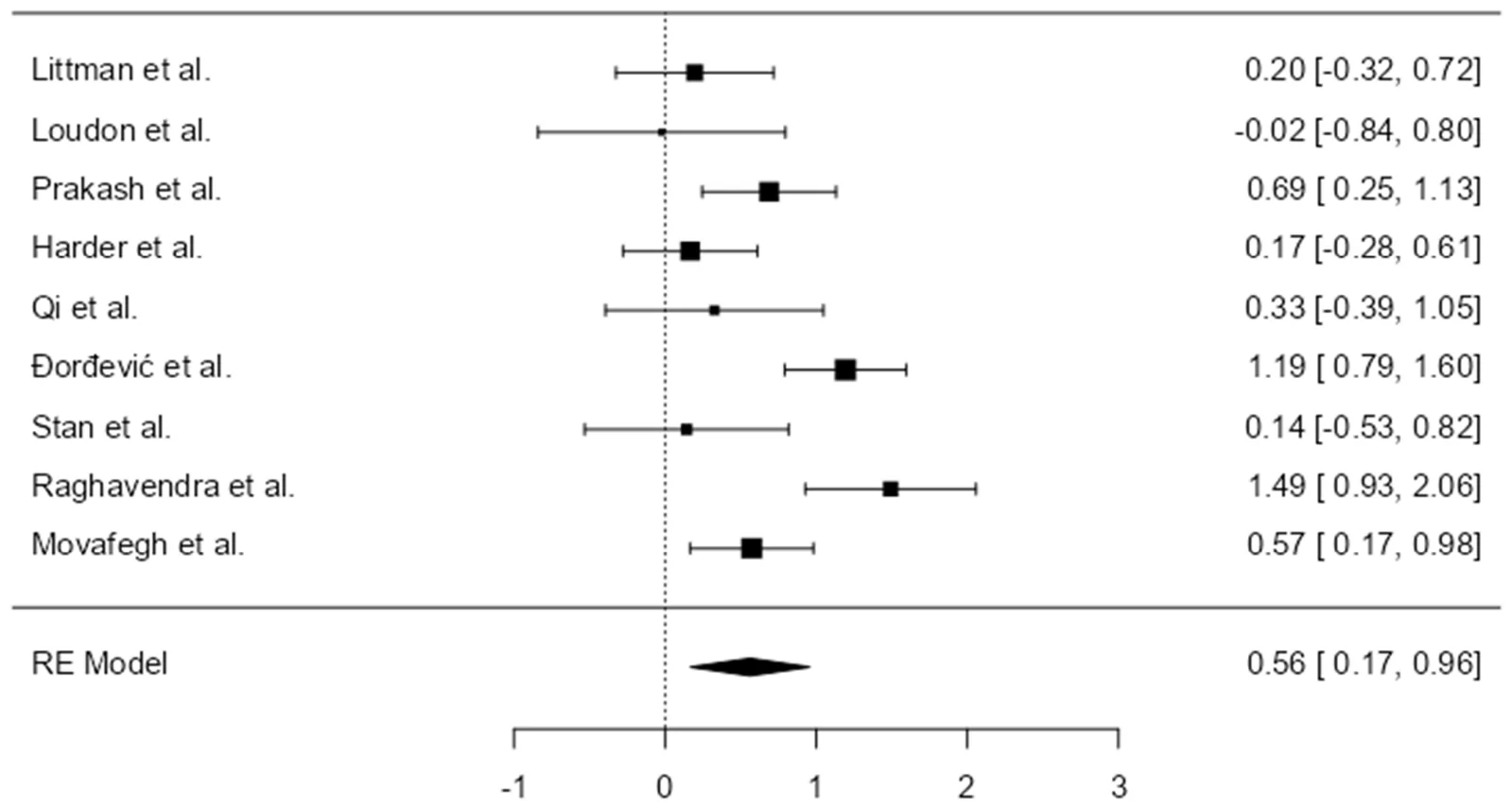
Forest plot of the primary meta-analysis evaluating the effect of yoga on health-related quality of life (HRQoL) in women with breast cancer. The analysis included nine independent randomized controlled trials and was performed using a random-effects model. Effect sizes are expressed as standardized mean differences (Hedges’ g) with corresponding 95% confidence intervals. Positive values indicate a beneficial effect of yoga compared with control conditions. The pooled analysis demonstrated a moderate and statistically significant improvement in HRQoL (Hedges’ g = 0.56, 95% CI: 0.17–0.96), although substantial between-study heterogeneity was observed (I^2^ = 72.4%).

To quantitatively synthesize the available evidence on HRQoL, a predefined meta-analysis was performed according to the eligibility and outcome selection criteria described in the Methods. Among the 18 RCT publications included in the narrative synthesis, 13 provided sufficient quantitative data to calculate standardized mean differences [17,29,32,34,37,38,40,41,42,45,46,50,51]. For the primary meta-analysis, quantitative synthesis was restricted to HRQoL outcomes. To preserve statistical independence and avoid double-counting participants, only one effect estimate from each independent study population was included. Studies reporting outcomes different from quality of life in study populations were not included in the pooled HRQoL estimate. Consequently, nine independent RCTs contributed to the primary quantitative synthesis [17,29,32,37,38,45,46,50,51]. Supplementary Table S5 summarizes the eligibility of the RCT publications for quantitative synthesis and provides the reasons for exclusion from the primary meta-analysis. The primary meta-analysis included nine independent RCTs [29,32,37,38,45,46,48,50,51] and evaluated the effect of yoga on HRQoL. The random-effects model showed a moderate and statistically significant improvement in HRQoL in favour of remotely delivered yoga (Hedges’ g = 0.56, 95% CI 0.17 to 0.96; *p* = 0.011). Between-study heterogeneity was substantial (I^2^ = 72.4%; τ^2^ = 0.181; Q(8) = 28.61, *p* < 0.001) (Figure 2). The 95% prediction interval ranged from −0.49 to 1.62, indicating considerable variability in the expected true effects across comparable settings and suggesting that a future comparable study could plausibly show little or no benefit. No study was identified as an outlier based on studentized residuals or as overly influential according to Cook’s distance.

Inspection of the individual study estimates showed variability in the magnitude of the treatment effects. The largest effects were observed in Raghavendra et al. [38] (Hedges’ g = 1.49, 95% CI: 0.93 to 2.06) and Đorđević et al. [51] (Hedges’ g = 1.19, 95% CI: 0.79 to 1.60). Both studies evaluated comprehensive yoga interventions delivered through relatively intensive programs in women undergoing active treatment or during BC survivorship. However, given the substantial overall heterogeneity and the limited number of independent studies, differences in intervention characteristics, participant populations, and clinical settings should be considered as possible sources of variability rather than established moderators of treatment effect. In contrast, Loudon et al. [48], which enrolled women with breast cancer-related lymphedema and assessed HRQoL using the LYMQOL, showed no significant difference between groups (Hedges’ g = −0.02, 95% CI: −0.85 to 0.81). This finding should likewise be interpreted cautiously and may reflect differences in the study population, intervention characteristics, or outcome assessment (Table S6).

An exploratory univariate mixed-effects meta-regression was first performed to assess whether publication year influenced the effect of yoga on HRQoL. No significant association was observed between publication year and the pooled effect size (β = −0.005, SE = 0.031, z = −0.15, *p* = 0.881; 95% CI: −0.066 to 0.056). Residual heterogeneity remained substantial (Q(8) = 27.66, *p* < 0.001; τ^2^ = 0.21; I^2^ = 75.0%), and publication year explained none of the between-study variance (R^2^ = 0%). However, these findings should be interpreted cautiously given the limited number of studies available for meta-regression.

Additional exploratory univariate meta-regression analyses examined WHO region, clinical setting, yoga type, delivery mode, and intervention duration. No statistically significant associations were identified. However, given the limited number of independent studies and sparse observations within several categories, these analyses were underpowered and should be interpreted as hypothesis-generating only. Non-significant findings should not be interpreted as evidence that these characteristics do not contribute to between-study heterogeneity.

No statistically significant evidence of funnel-plot asymmetry was detected according to Begg and Mazumdar’s rank correlation test (τ = −0.222, *p* = 0.477) or Egger’s regression test (intercept = −1.228, *p* = 0.259). The trim-and-fill procedure imputed one potentially missing study, and Rosenthal’s fail-safe N was 133. However, given the small number of independent studies, these analyses have limited statistical power and should be considered exploratory; therefore, publication bias and small-study effects cannot be reliably excluded.

### 3.5. Psychological Outcomes

Psychological outcomes were assessed in 14 publications (53.8%), including anxiety (10 publications, 38.5%), depression (8 publications, 30.8%), perceived stress (4 publications, 15.4%), sleep quality (5 publications, 19.2%), cognitive function (3 publications, 11.5%), mental well-being (2 publications, 7.7%), self-esteem/confidence (1 publication, 3.8%), and fear of recurrence (1 publication, 3.8%). Findings suggested potential benefits across several psychological domains, although results were heterogeneous across studies, intervention characteristics, clinical contexts, and assessment instruments.

Anxiety was the most frequently investigated psychological outcome. Significant reductions were reported particularly in women undergoing active treatment. Raghavendra et al. demonstrated substantial improvements in anxiety symptoms measured by the State-Trait Anxiety Inventory (STAI), with significant reductions maintained across different treatment phases [38,39]. Similarly, Kovačič et al. reported significant reductions in anxiety following a yoga-based relaxation intervention (all *p* < 0.0005) [41,42], while Lengacher et al. observed a significant reduction in anxiety after a mindfulness-based yoga programme (*p* = 0.03; d = 0.72) [53]. More recently, Ferrigno Guajardo et al. reported sustained reductions in GAD-7 scores at 2, 5, and 8 months after intervention (*p* < 0.001 to 0.001) [47]. Conversely, no significant changes in anxiety were observed in Komatsu et al. and Qi et al., highlighting variability across populations and intervention protocols [45,55]. Among quasi-experimental studies, Flanagan et al. observed a progressive reduction in anxiety symptoms, with GAD scores decreasing from baseline to week 4 (8.24 ± 5.03 to 5.17 ± 3.22) [44].

Depressive symptoms were assessed in eight publications. The strongest evidence came from Raghavendra et al. [38,39], who reported significant reductions in depression measured by the Beck Depression Inventory during surgery, radiotherapy, and chemotherapy phases. Lengacher et al. also demonstrated significant improvement in depressive symptoms (*p* < 0.01; d = 0.85) [53]. Improvements were additionally reported by Prakash et al. during chemotherapy cycles [36], whereas Lanctôt et al. and Komatsu et al. did not observe significant changes in depression scores [34,55].

Perceived stress was evaluated in four publications. Lengacher et al. reported a significant reduction in perceived stress following yoga intervention (*p* = 0.004; d = 0.84), accompanied by improvements in mindfulness-related outcomes [53]. Similarly, Kovačič et al. observed significant reductions in psychological distress and perceived stress at both short-term and follow-up assessments (*p* = 0.0005) [40]. Conversely, Qi et al. did not identify significant changes in perceived stress [45].

Sleep quality was assessed in five publications, with overall findings suggesting potential benefits of yoga on sleep-related symptoms. Lengacher et al. reported significant improvement in daytime sleep dysfunction (*p* = 0.01; d = 0.65 [53]), while Ferrigno Guajardo et al. demonstrated significant improvement in insomnia symptoms at multiple follow-up points (*p* = 0.004, 0.032, and 0.002) [47]. Đorđević et al. also reported significant improvement in sleep quality measured by the Pittsburgh Sleep Quality Index (*p* = 0.007) [51].

Cognitive outcomes were explored in three publications. Neville et al. reported significant improvements in episodic memory and executive function after an eight-week yoga intervention (both *p* = 0.009) [54], whereas Lengacher et al. observed improvements in cognitive function, particularly language abilities (*p* = 0.03) [53]. Conversely, Komatsu et al. found no significant improvement in overall cognitive function, although cognitive fatigue was reduced [55].

Mental well-being was evaluated in two publications. Flanagan et al. reported improved Warwick-Edinburgh Mental Well-being Scale scores after four weeks of yoga practice (26.31 ± 5.01 to 28.69 ± 4.09) [44]. Lengacher et al. also reported improvements in emotional well-being domains of the SF-36 [53].

Fear of recurrence and self-esteem were investigated in single publications. Lengacher et al. observed significant reductions in overall fear of recurrence (*p* = 0.03; d = 0.74) and recurrence-related concerns (*p* = 0.05; d = 0.60) [53]. Kovačič et al. reported significant improvements in self-esteem at both post-intervention and follow-up assessments (*p* < 0.0005) [41].

### 3.6. Physical Outcomes

Physical and symptom-related outcomes were assessed in 16 publications (61.5%). CRF was the most frequently investigated physical outcome (9 studies, 34.6%), followed by lymphedema-related outcomes (4 studies, 15.4%), mobility and physical function outcomes (3 studies, 11.5%), pain (3 studies, 11.5%), treatment-related symptoms/toxicity (3 studies, 11.5%), and anthropometric measures (1 study, 3.8%). Overall, remotely delivered yoga interventions were associated with improvements in several physical domains, particularly fatigue severity, functional capacity, and selected treatment-related symptoms; however, findings remained heterogeneous depending on the clinical context, intervention format, and outcome measures adopted.

CRF represented the most consistently investigated physical symptoms. Across randomized and non-randomized studies, remotely delivered yoga interventions generally showed beneficial effects on fatigue severity and its interference with daily activities. Lengacher et al. reported significant reductions in fatigue severity and fatigue-related interference after a mindfulness-based yoga intervention (*p* = 0.002 and *p* = 0.03, respectively) [53], while Neville et al. observed significant improvements in overall fatigue measured by the Revised Piper Fatigue Scale (*p* = 0.005), including sensory fatigue (*p* = 0.007) and cognitive/mood fatigue (*p* = 0.002) [54]. Similarly, Komatsu et al. identified a significant reduction in cognitive fatigue (*p* = 0.01), although no significant improvements were observed in physical, affective, or overall fatigue domains [55]. Conversely, Littman et al., Stan et al. and Qi et al. did not demonstrate significant reductions in fatigue-related outcomes, suggesting that effects may depend on baseline symptom burden and intervention characteristics [29,32,45].

Lymphedema-related outcomes were evaluated in four studies, mainly among BC survivors with breast cancer-related lymphedema. Loudon et al. demonstrated that yoga was feasible and safe, with improvements in lymphedema-specific QoL at the end of the intervention (*p* = 0.038), although no significant differences were observed in arm volume or extracellular fluid compared with controls [48]. Similarly, Harder et al. found no additional benefit of yoga on arm volume reduction or upper-limb symptoms [46]. More recently, Movafegh et al. reported significant improvements in lymphedema-related impairment, including reductions in limb volume (*p* = 0.042) and improvements in LLIS total and functional/physical domains (*p* = 0.006–0.017), suggesting a potential role of yoga as a supportive strategy for managing functional consequences of lymphedema [50].

Mobility and physical function outcomes were investigated in four studies. Loudon et al. reported improvements in lumbo-pelvic posture and shoulder abduction strength, although range of motion did not significantly change [48]. Faravel et al. observed improvements in flexibility, measured by the fingertip-to-floor test, with effects maintained during follow-up [56]. Furthermore, Anestin et al. reported improvements in motivation toward physical activity after an eight-week intervention (*p* < 0.001) [35], while Neville et al. observed increased moderate-to-vigorous physical activity levels following yoga practice (mean difference = 93.1 ± 38.8 min/week; *p* = 0.03) [54].

Pain outcomes were assessed in three studies, with mixed but overall promising findings. Faravel et al. reported a significant reduction in hormone therapy-associated osteo-articular pain, with median pain scores decreasing from 6 to 4 after intervention (*p* < 0.01), and clinically meaningful improvement achieved by more than half of participants [56]. In contrast, Qi et al. and Lengacher et al. did not observe significant changes in pain, suggesting that yoga-related benefits may be more evident for specific pain conditions rather than general pain intensity [45,53].

Treatment-related symptoms and toxicity were investigated mainly among women undergoing active cancer treatment. Raghavendra et al. reported reductions in chemotherapy-related toxicity and nausea/vomiting symptoms following yoga intervention, including significant decreases in post-chemotherapy nausea frequency and severity (*p* = 0.01–0.003) [38]. Similarly, Prakash et al. observed reductions in chemotherapy-related symptom burden across treatment cycles [37].

Anthropometric outcomes were assessed in one randomized study [32]. Littman et al. reported a significant reduction in waist circumference among breast cancer survivors participating in a yoga intervention (−3.1 cm; 95% CI −5.7 to −0.4), whereas no significant changes were observed in body weight, BMI, or hip circumference. These findings suggest a possible effect of yoga on selected anthropometric parameters; however, evidence remains limited and further studies are required to clarify whether yoga may contribute to long-term changes in body composition.

### 3.7. Feasibility, Adherence and Safety

Feasibility, adherence, and safety were among the most frequently reported outcomes related to the intervention implementation, being assessed in 15 publications (57.7%). Overall, remotely delivered yoga interventions appeared feasible across different BC populations and delivery formats, with recruitment, retention, adherence, and acceptability showing substantial variability depending on intervention intensity, level of supervision, and study design.

Recruitment and retention outcomes were generally favorable in most RCTs. Stan et al. reported a recruitment rate of 68% and a completion rate of 74% in the yoga group, supporting the feasibility of a home-based remotely delivered programme [29]. Similarly, Qi et al. observed a high retention rate (93.3%), despite a moderate recruitment rate (55.6%) [45]. Neville et al. demonstrated particularly strong feasibility indicators for an eight-week online yoga intervention, with an enrolment rate of 66.7%, low attrition (5.3%), and adherence exceeding the predefined feasibility threshold (83.7%) [54]. Conversely, some studies reported higher attrition or lower completion rates, such as Đorđević et al., where loss to follow-up was approximately one-third of participants in both intervention and control groups [51], and Zok et al., where only 31.7% of enrolled participants completed the intervention [52].

Adherence to yoga practice varied considerably across studies, reflecting differences in intervention structure and participant requirements. Higher adherence was observed in interventions incorporating regular supervision and structured home practice. Faravel et al. reported an adherence rate of 83%, with 75% of participants attending all supervised sessions and a median of 74 home practice sessions completed [56]. Neville et al. similarly reported high session attendance, with participants completing an average of 14 out of 16 classes [54]. In contrast, adherence was more moderate in less supervised programmes, such as Stan et al. (39%) [29] and Cadmus-Bertram et al. (58% of the prescribed programme) [33], although the latter reported that 59% of participants completed at least half of the scheduled sessions. Littman et al. also demonstrated good long-term engagement, with mean participation of 19.6 supervised classes and 55.8 home sessions over 26 weeks [32].

Beyond adherence, several studies explored participant acceptability and perceived usefulness of remotely delivered yoga. Komatsu et al. reported high acceptability, with most participants considering the intervention interesting (88.9%), useful (72.2%), and expressing willingness to continue yoga practice after study completion (72.2%) [55]. Winters et al. similarly highlighted the convenience and accessibility of video-based yoga, although some participants suggested the need for more detailed guidance on relaxation techniques [43]. Positive perceptions were also reported by Lanctôt et al., where participants rated yoga components favorably despite moderate attendance [34].

Safety outcomes were reported in 9 studies (34.6%) [32,35,43,45,46,48,54,55,56]. Among these publications, no serious adverse events attributable to yoga practice were reported. However, adverse-event monitoring and reporting were not consistently described across studies; therefore, the absence of reported serious adverse events should not be interpreted as establishing the overall safety of remote or home-based yoga interventions.

## 4. Discussion

The aim of this systematic review was to provide a comprehensive summary of the currently available literature exploring the effects of remotely delivered yoga on HRQoL and secondary psychological and physical outcomes, as well as feasibility, adherence, and safety, in women with BC. The review included 26 publications representing 19 independent cohorts, comprising randomized and non-randomized evidence. The primary meta-analysis focused on HRQoL, while secondary outcomes were primarily synthesized narratively. Overall, findings suggested potential benefits across several psychological and physical domains, including anxiety, depression, stress, sleep, cognitive function, mental well-being, fear of disease recurrence, self-esteem, CRF, mobility and physical function, treatment-related symptoms, and anthropometric measures, although results were heterogeneous across studies. Feasibility, adherence, and acceptability were generally favorable in several interventions, although these outcomes varied according to study and delivery characteristics. Among studies reporting safety outcomes, no serious adverse events attributable to yoga were reported. A clear direction could not be established for lymphedema-related outcomes and pain. As for HRQoL, the meta-analysis performed on nine independent RCTs showed a moderate and statistically significant improvement among participants receiving remote yoga compared with controls (Hedges’ g = 0.56). However, between-study heterogeneity was moderate-to-substantial (I^2^ = 72.4%), and the 95% prediction interval (−0.49 to 1.62) crossed the null, indicating that the magnitude and potentially the presence of benefit may vary across future comparable populations and intervention settings. Differences in delivery format may have contributed to this variability. In particular, the interventions included ranged from synchronous, instructor-supervised programs delivered through videoconferencing platforms to asynchronous or self-directed approaches based on DVDs, videos, audio recordings, or other home-based materials, as well as hybrid programs combining remote and face-to-face components. Real-time supervision may provide greater opportunities for individualized guidance, correction of practice, and participant engagement, whereas asynchronous approaches may offer greater flexibility and accessibility. However, in our study, the available evidence did not allow to state conclusions regarding the comparative effectiveness of these delivery models. Exploratory meta-regression analyses did not identify statistically significant associations between publication year, geographical region, clinical setting, yoga type, intervention characteristics, and the pooled effect size. However, given the limited number of independent studies, these analyses were underpowered and non-significant findings cannot be interpreted as evidence that these factors do not contribute to between-study heterogeneity. A potentially relevant aspect for clinical implementation is the role of supervision in supporting adherence. Several interventions reporting relatively high adherence incorporated regular instructor contact or real-time supervision, whereas adherence was more variable in less supervised, self-directed programs. Real-time interaction may facilitate engagement, provide feedback, and allow adaptations according to participants’ needs. Nevertheless, because supervised and unsupervised delivery formats were not directly compared and adherence was defined differently across studies, these observations should be considered hypothesis-generating rather than evidence of a causal effect of supervision on adherence. These findings are partially consistent with existing literature on conventionally supervised yoga. A systematic review and meta-analysis of 18 studies reported reductions in fatigue and negative emotional symptoms and improvements in sleep and quality of life in patients with breast cancer, although no significant changes were observed for anxiety and depression [57]. A meta-analysis of 21 RCTs reported significant reductions in depressive symptoms associated with yoga interventions in BC patients, although effects varied across interventions [58]. The systematic review by Kumari et al. reported significant short-term improvements in anxiety, depression, emotional function, and fatigue levels related to yoga interventions, independently by yoga style and intervention duration. Furthermore, regular practice of yoga had also positive impacts on cognition, pain, gastrointestinal outcomes, swelling, and shoulder strength [59].

A recent dose–response meta-analysis identified the optimal weekly dose of yoga exercise (220 min/week) to improve fatigue, sleep quality, and QoL in patients with breast cancer [31]. The Authors, however, invite practitioners to pay attention to the therapeutic stage and the age of the patients in order to make yoga-based intervention effective. In fact, they found that yoga was less effective in reducing fatigue during treatment than in the post-treatment period, while its effects on quality of life and sleep remained stable. Furthermore, with increasing age, the benefits of yoga interventions on fatigue and sleep quality decrease, while the benefits on quality of life increase.

To the Authors’ knowledge, no systematic reviews and meta-analyses have been published so far specifically examining remotely delivered yoga interventions in women with BC. Komariah and colleagues analyzed remote-based mindfulness interventions on physical symptoms in cancer survivors [60]. Their findings suggested reductions in fatigue and sleep disturbance and improvement in physical function, while no significant effect on pain was observed. These findings are broadly consistent with some of the secondary outcomes identified in the present review; however, direct comparison is limited because Komariah et al. included different cancer populations and evaluated remote mindfulness interventions rather than specifically yoga-based programs.

However, several important limitations of this systematic review should be considered when interpreting these findings. First, despite the inclusion of 26 publications, these represented only 19 independent cohorts, and the primary HRQoL meta-analysis included nine independent RCTs. The limited number of independent studies reduced the statistical power of moderator analyses and precluded robust subgroup comparisons, which would have been particularly informative given the substantial heterogeneity of the interventions. Furthermore, several secondary outcomes were evaluated in only a small number of studies, which did not allow subgroup comparisons. Moreover, though the majority of methodological quality aspects were respected, concerns remain about participant, supervisor and assessor blinding, which were not reported in 100, 94 and 61% of the studies, respectively. Therefore, the pooled HRQoL estimate and the findings for secondary outcomes should be interpreted cautiously, and additional adequately powered RCTs are needed to strengthen the evidence base. Second, considerable clinical and methodological heterogeneity was observed across studies, including differences in intervention duration, delivery mode, degree of supervision, participant clinical status, outcome measures, and assessment timing. In particular, the inclusion of synchronous, asynchronous, home-based, and hybrid delivery models limits the extent to which the pooled findings can be attributed to a specific form of remote yoga delivery and currently prevents identification of the most effective delivery strategy. Third, the small number of long-term interventions limits conclusions regarding the persistence of intervention effects over time. Further studies should also evaluate the persistence of the other effects after treatment, and the motivation to continue practicing yoga after the intervention ends.

Fourth, evidence for several secondary psychological and physical outcomes was derived partly from non-randomized or single-arm studies. Improvements observed within these studies cannot be interpreted as causal effects of yoga because they may reflect temporal changes, concurrent treatments, regression to the mean, or other uncontrolled factors. Accordingly, greater evidential weight should be given to controlled between-group findings from randomized trials.

Finally, safety outcomes were reported in only a subset of publications, and adverse-event monitoring procedures were not consistently described. Therefore, although no serious yoga-related adverse events were reported in these studies, the available evidence is insufficient to establish the overall safety of remote or home-based yoga interventions.

Despite these limitations, the restriction of the primary meta-analysis to independent RCTs reporting HRQoL minimized the risk of double-counting participants and provided a methodologically consistent estimate of the pooled effect. The broader inclusion of randomized and non-randomized evidence also allowed relevant aspects of remote delivery, including feasibility, adherence, safety, and secondary psychological and physical outcomes, to be comprehensively examined.

## 5. Conclusions

This review suggests that remote and home-based yoga interventions may represent a promising supportive approach for women with breast cancer, with the primary meta-analysis showing a moderate improvement in HRQoL. Potential benefits were also observed across several psychological and physical outcomes, although the evidence was heterogeneous. Given the limited number of independent studies, substantial between-study heterogeneity, and variability in intervention delivery, these findings should be interpreted with caution. Further adequately powered, long-term RCTs using standardized intervention and reporting protocols are needed to confirm these findings and identify the most appropriate delivery modalities according to patients’ clinical characteristics and needs.

## Figures and Tables

**Table 2:** Outcomes changes and assessment methods used in the selected studies.

Ref. No.	QoL	Psychological Outcomes Assessment Tool; Change						Physical Outcomes Assessment Tool; Change				
		Anxiety; Depression	Stress	Sleep	Cognitive Functions	Mental Well-Being	Fear of Recurrence; Self Esteem	Fatigue; Pain	TX Side Effects, Toxicity	Anthropometric Measurements	Mobility, Physical Activity	Lymphoedema Related Outcomes
[51]	EORTC QLQ-C30/BR23; ↑			PSQI; ↑* (*p* = 0.007).								
[29]	FACT-B; ↑* functional well-being domain (*p* = 0.005).							MFSI-SF; ↔				
[48]	LYMQOL; ↑* at the end of the intervention (*p* = 0.038); ↑ at 1-month follow-up.											↔ Arm volume at week 8; ↑* at week 12 (*p* = 0.032).
[49]												↑* Posture/strength: lumbo-pelvic posture (*p* = 0.023) and shoulder abduction strength (*p* = 0.042 and 0.045). ↔ ROM
[36]		ADSS; ↔ anxiety within-group; ↓* anxiety between-group (cycles II, III, and VI); ADSS; ↓* depression between groups (cycles II–VI; *p* = 0.02 to < 0.001).	ADSS; ↑* between groups during cycle III (*p* = 0.01)									
[37]	QOL-C30; ↑* during the 2nd–6th chemotherapy cycles (*p* < 0.01).			QOL-C30; ↑* in the fifth cycle (*p* = 0.01)				QOL-C30; ↓* during chemotherapy cycles II–VI (*p* = 0.001 to <0.001).	QOL-C30; ↓* only during cycle III (*p* = 0.02).			
[38]	FLIC; ↑* (*p* < 0.001)	STAI; ↓* anxiety (*p* < 0.001); BDI; ↓* depression.							↓* MANE nausea frequency and severity, anticipatory nausea/vomiting symptoms (*p* = 0.01 to 0.003).			
[39]	FLIC; ↑* after surgery (*p* = 0.01), during RT (*p* < 0.001) and during CT (*p* < 0.001)	STAI; ↓* anxiety after surgery (*p* = 0.04), before RT (*p* = 0.005), during RT (*p* = 0.009), and during and after CT (*p* < 0.001); BDI; ↓*depression after surgery (*p* = 0.01), before RT (*p* = 0.007), after RT (*p* < 0.001), before CT (*p* = 0.02), and after CT (*p* = 0.002).							WHO Toxicity Criteria; ↓* chemotherapy-induced toxicity (*p* = 0.01).			
[40]			GHQ-12, RSCL, PSS; ↓* psychological distress and perceived stress at 1- and 4-week follow-up (*p* = 0.0005).									
[41]		STAI (Y-1/Y-2); ↓*anxiety after relaxation training and at follow-up (all *p* < 0.0005).					RSE; ↑* self-esteem at 1- and 4-week follow-up (*p* < 0.0005).					
[42]		STAI (Y-1/Y-2); ↓*anxiety after relaxation training and at follow-up (all *p* < 0.0005).										
[32]	FACT-G; ↔ ↑* in participants attending ≥24 (*p* < 0.05).							MFSI-SF; ↔ fatigue.		↓ Waist circumference (−3.1 cm; 95% CI −5.7 to −0.4). ↔ weight, BMI, or hip circumference.	↔ Non-yoga physical activity (MET-h/week).	
[34]		STAI-Y; ↔ anxiety; BDI-II; ↔ depression.										
[35]								GPF; ↔ fatigue; ↑ in the waitlist group (group × time interaction: *p* = 0.03). MOT; ↑* motivation after 8 weeks (*p* < 0.001).				
[45]	FACT-B; ↑* (*p* = 0.006).	HADS; ↔ anxiety; HADS; ↔ depression.	PSS ↔	MDASI-C ↔				↔ MDASI-C fatigue and pain.				
[46]	FACT-B+4 ↑* among patients receiving adjuvant chemotherapy (*p* < 0.001).											↔ Arm volume. ↔ Arm function/upper limb symptoms.
[50]	LLIS; ↑* physical and functional domains and total score (all *p* < 0.001).											↓* volume (*p* = 0.042; within-group *p* < 0.001) LLIS/QoL; ↑* lymphoedema-related QoL, including total LLIS score and functional/physical domains (*p* = 0.006–0.017; within-group *p* < 0.001).
[47]		GAD-7; ↓* anxiety at 2, 5, and 8 months (*p* < 0.001 to 0.001); PHQ-9; ↓* depression, particularly at 2 and 8 months (*p* < 0.001 and *p* = 0.019) and at 5 months (*p* = 0.024).		ISI; ↑* sleep/insomnia symptoms at 2, 5, and 8 months (*p* = 0.004, 0.032, and 0.002).		CWS; ↓* at 2 and 5 months (*p* < 0.001 to 0.014); MAAS; ↑*at 2, 5, and 8 months (*p* = 0.002 to 0.007).		FACIT-F; ↓*at 2 months, with sustained effects at 5 months (*p* = 0.007 and *p* = 0.024).				
[52]	↑* self-rated health.											
[43]												
[55]	FACT-B; ↔	HADS; ↔ anxiety; HADS; ↔ depression.			CFQ; ↔			CFS; ↓* cognitive fatigue domain (*p* = 0.01); ↔ physical, affective, or overall fatigue.				
[53]	SF36; ↑* vitality (*p* = 0.02), emotional well-being (*p* = 0.03), general health (*p* = 0.01), physical health (*p* = 0.03)	STAI; ↓* anxiety (*p* = 0.03); CES-D; ↓* depression (*p* < 0.01)	PSS; ↓* perceived stress (*p* = 0.004)	PSQI; ↓* daytime sleep dysfunction (*p* = 0.01)	ECog; ↑* cognitive function, specifically language abilities (*p* = 0.03)			FSI; ↓* severity and interference of fatigue with daily activities (*p* = 0.002, *p* = 0.03); CARS; ↓* overall fear (*p* = 0.03) problems associated with recurrence concerns (*p* = 0.05); BPI; ↔ pain.				
[56]								VAS; ↓* osteo-articular pain, maintained at follow-up (*p* < 0.01).			Fingertip-to-floor test ↑ Flexibility.	
[33]												
[34]		GAD; ↓ Anxiety (mean score: 8.24 to 5.17).				WEMWBS; ↑ Mental well-being (mean score: 26.31 to 28.69).						
[54]					Picture Sequence Memory Test and Task Switching Test; ↑* episodic memory and executive function (reaction time) (both *p* = 0.009).			PFS-Revised ↓* overall fatigue (*p* = 0.005), sensory fatigue (*p* = 0.007) and cognitive/mood fatigue (*p* = 0.002).			↑* MVPA (*p* = 0.03).	

↑ increase; ↓ decrease; ↔ no change; * statistically significant change in randomized controlled studies; ↑ increase; ↓ decrease; ↔ no change; * statistically significant change in non-randomized studies.

## Data Availability

No new data were created or analyzed in this study. Data sharing is not applicable to this article.

## Supplementary Materials

The following supporting information can be downloaded at: https://www.mdpi.com/article/10.3390/healthcare14183040/s1, Figure S1: Sensitivity analysis. Forest plot of the sensitivity analysis including all eligible effect sizes across all reported outcomes (k = 13), including multiple correlated outcomes from the same randomized controlled trial. The pooled effect was estimated using a random-effects model; Figure S2. PRISMA 2020 Main Checklist [22]; Figure S3. PRISMA Abstract Checklist [22]. Table S1: Complete electronic search strategies; Table S2: Outcome Measures Assessed in the Included Studies; Table S3: JBI Critical Appraisal of the Included Randomized Controlled Trials (RCTs); Table S4: JBI Critical Appraisal of the Included Quasi-Experimental Studies; Table S5: Eligibility of Randomized Controlled Trials for Quantitative Synthesis; Table S6: Data extraction and effect-size calculation for studies included in the primary HRQoL meta-analysis.

## Abbreviations

The following abbreviations are used in this manuscript:

BC	Breast Cancer
BDI	Beck’s Depression Inventory
BMI	Body Mass Index
BPI	Brief Pain Inventory
CARS	Conflict Anxiety Response Scale
CFQ	Cognitive Failures Questionnaire
CFS	Clinical Frailty Scale
CI	Confidence Interval
CRF	Cancer-related fatigue
CT	Chemotherapy treatment
CWS	Cancer Worry Scale
FACT-B	Functional Assessment of Cancer Therapy-Breast cancer
FACT-G	Functional Assessment of Cancer Therapy-General
ECOG	Eastern Cooperative Oncology Group
EORTC	European Organisation for Research and Treatment of Cancer
FACIT-F	Functional Assessment of Chronic Illness Therapy-Fatigue
FLIC	Functional Living Index for Cancer
GAD	General Anxiety Disorder
GHQ	General Health Questionnaire
GPF	Global Perception of Fatigue
HADS	Hospital Anxiety and Depression Scale
HRQoL	Health-related quality of life
ISI	Insomnia Severity Index
JBI	Joanna Briggs Institute
LLIS	Lymphedema Life Impact Scale
LYMQOL	Lymphoedema Quality of Life Questionnaire
MANE	Morrow Assessment of Nausea and Emesis
MDASI	MD Anderson Symptom Inventory
MET	Metabolic Equivalent
MFSI-SF	Multidimensional Fatigue Symptom Inventory-Short Form
MOT	Motricity Index
MVPA	moderate-to-vigorous physical activity
PFS	Pittsburgh Fatigability Scale
PHQ	Patient Health Questionnaire
PSS	Perceived Stress Scale
PSQI	Pittsburgh Sleep Quality Index
QLQ-C30	Quality of Life Questionnaire Core 30
RCT	Randomized controlled study
ROM	Range of Motion
RSCL	Rotterdam Symptom Checklist
RSS	Rosenberg Self-Esteem Scale
RT	Radiotherapy Treatment
SD	Standard Deviation
SE	Standard Error
STAI	State-Trait Anxiety Inventory
VAS	Visual Analogue Scale
WHO	World Health Organization
WEMWBS	Warwick-Edinburgh Mental Well-being Scale
