# Supplementary material for: Effects of Remotely Delivered Yoga Interventions on Quality of Life and Symptom Burden in Women with Breast Cancer: A Systematic Review and Meta-Analysis
Source: Healthcare (Basel). 2026 Sep 16;14(18):3040. doi: 10.3390/healthcare14183040 (PMC13606698; doi:10.3390/healthcare14183040)
Supplement: Supplementary file 1 [file healthcare-14-03040-s001.zip › Figure S1.pdf]

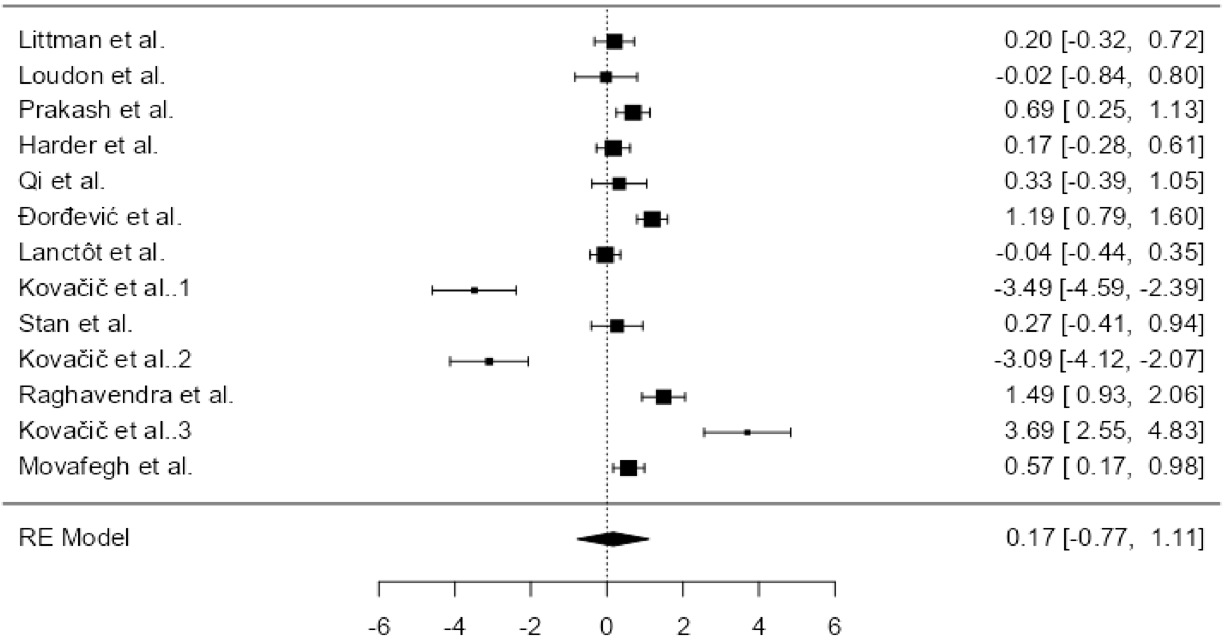

Supplementary Figure S1. Sensitivity analysis. Forest plot of the sensitivity analysis including all eligible effect sizes across all reported outcomes (k = 13), including multiple correlated outcomes from the same randomized controlled trial. The pooled effect was estimated using a random-effects model.
