# Supplementary material for: Effects of Remotely Delivered Yoga Interventions on Quality of Life and Symptom Burden in Women with Breast Cancer: A Systematic Review and Meta-Analysis
Source: Healthcare (Basel). 2026 Sep 16;14(18):3040. doi: 10.3390/healthcare14183040 (PMC13606698; doi:10.3390/healthcare14183040)
Supplement: Supplementary file 1 [file healthcare-14-03040-s001.zip › Table S1.pdf]

Supplementary Table S1. Complete electronic search strategies.

The table reports the complete search strings applied to each database included in the systematic literature search: a) MEDLINE (via PubMed), b) Scopus, c) Web of Science, and the d) Cochrane Library. Search strategies were adapted according to the specific syntax and controlled vocabulary of each database while maintaining the same three conceptual domains: (1) breast cancer, (2) yoga interventions, and (3) remote/digital delivery modalities.

a) MEDLINE

#1	Breast cancer[tiab] OR breast carcinoma[tiab] OR cancer*[tiab] OR neoplasm*[tiab] OR tumor*[tiab] OR tumour*[tiab] OR malignan*[tiab] OR carcinoma*[tiab] OR oncolog*[tiab] OR (breast*[tiab] AND (cancer[tiab] OR tumor*[tiab] OR tumour*[tiab] OR malignan*[tiab])) OR (mammary[tiab] AND (cancer[tiab] OR tumor*[tiab] OR tumour*[tiab] OR malignan*[tiab])) OR "Breast Neoplasms"[Mesh] OR "Breast Diseases"[Mesh] OR "Neoplasms"[Mesh] OR "Carcinogenesis"[Mesh] OR "Neoplastic Processes"[Mesh]
#2	yoga[tiab] OR yogi*[tiab] OR asana[tiab] OR Iyengar[tiab] OR Ashtanga[tiab] OR Bikram[tiab] OR hatha[tiab] OR vinyasa[tiab] OR tibetan[tiab] OR kundalini[tiab] OR nidra[tiab] OR yoga[MeSH] OR (yoga[tiab] AND (class*[tiab] OR group*[tiab] OR lesson*[tiab] OR session*[tiab] OR practice*[tiab]))
#3	online[tiab] OR web-based[tiab] OR web[tiab] OR Internet-Streamed[tiab] OR internet[tiab] OR stream*[tiab] OR digital*[tiab] OR home-based[tiab] OR home based[tiab] OR home-programme[tiab] OR home[tiab] OR at home[tiab] OR domiciliary[tiab] OR videoconference[tiab] OR video-based[tiab] OR app-based[tiab] OR application*[tiab] OR iPad[tiab] OR remote[tiab] OR mobile[tiab] OR mobile app*[tiab] OR video*[tiab] OR device-led[tiab] OR tele-yoga[tiab] OR telehealth[tiab] OR telemedicine[tiab] OR e-health[tiab] OR Provider-Led[tiab] OR supervised[tiab] OR operator[tiab] OR instructor*[tiab] OR instructor-led[tiab] OR guided[tiab] OR live[tiab] OR unsupervised[tiab] OR self-guided[tiab] OR tutorial[tiab] OR console[tiab] OR “active video-game”[tiab] OR "fitness game"[tiab] OR exergame[tiab] OR gamification[tiab] OR wii[tiab] OR teacher*[tiab] OR coach*[tiab] OR trainer*[tiab] OR tutor*[tiab] OR guru*[tiab] OR guide*[tiab]

b) SCOPUS

#1	TITLE-ABS-KEY("breast cancer" OR "breast carcinoma" OR cancer* OR neoplasm* OR tumor* OR tumour* OR malignan* OR carcinoma* OR oncolog* OR (breast AND (cancer OR tumor* OR tumour* OR malignan*)) OR (mammary AND (cancer OR tumor* OR tumour* OR malignan*)) OR "breast neoplasm*" OR "breast disease*" OR neoplasm* OR carcinogenesis OR "neoplastic process*")

**#2**	TITLE-ABS-KEY(yoga OR yogi* OR asana* OR Iyengar OR Ashtanga OR Bikram OR hatha OR vinyasa OR tibetan OR kundalini OR (yoga AND class* OR group* OR lesson* OR session* OR practice*))
**#3**	TITLE-ABS-KEY(online OR “web-based” OR web OR “internet-streamed” OR internet OR stream* OR digital* OR “home-based” OR “home based” OR “home-programme” OR home OR “at home” OR domiciliary OR videoconference OR “video-based” OR “app-based” OR application* OR iPad OR remote OR mobile OR “mobile app*” OR video* OR “device-led” OR “tele-yoga” OR telehealth OR telemedicine OR e-health OR “provider led” OR supervised OR operator OR instructor* OR “instructor-led” OR guided OR live OR unsupervised OR “self-guided” OR tutorial OR console OR “active video-game” OR "fitness game" OR exergame OR gamification OR wii OR teacher* OR coach* OR trainer* OR tutor* OR guru* OR guide*)

c) WEB OF SCIENCE

**#1**	TS=("breast cancer" OR "breast carcinoma" OR cancer* OR neoplasm* OR tumor* OR tumour* OR malignan* OR carcinoma* OR oncolog* OR (breast AND (cancer OR tumor* OR tumour* OR malignan*)) OR (mammary AND (cancer OR tumor* OR tumour* OR malignan*)) OR "breast neoplasm*" OR "breast disease*" OR neoplasm* OR carcinogenesis OR "neoplastic process*")
**#2**	TS=(yoga OR yogi* OR asana* OR Iyengar OR ashtanga OR bikram OR hatha OR vinyasa OR tibetan OR kundalini) OR (yoga AND (class* OR group* OR lesson* OR session* OR practice*))
**#3**	TS=(online OR “web-based” OR web OR “internet-streamed” OR internet OR stream* OR digital* OR “home-based” OR “home based” OR “home-programme” OR home OR “at home” OR domiciliary OR videoconference OR “video-based” OR “app-based” OR application* OR iPad OR remote OR mobile OR “mobile app*” OR video* OR “device-led” OR “tele-yoga” OR telehealth OR telemedicine OR e-health OR “provider led” OR supervised OR operator OR instructor* OR “instructor-led” OR guided OR live OR unsupervised OR “self-guided” OR tutorial OR console OR “active video-game” OR "fitness game" OR exergame OR gamification OR wii OR teacher* OR coach* OR trainer* OR tutor* OR guru* OR guide*)

d) COCHRANE

**#1**	("breast cancer" OR "breast carcinoma" OR cancer* OR neoplasm* OR tumor* OR tumour* OR malignan* OR carcinoma* OR oncolog* OR (breast AND (cancer OR tumor* OR tumour* OR malignan*)) OR (mammary AND (cancer OR tumor* OR tumour* OR malignan*)) OR (breast NEXT neoplasm*) OR (breast NEXT disease*) OR carcinogenesis OR (neoplastic NEXT process*))<br>MeSH descriptor: [Breast Neoplasms] explode all trees

#**2**	(yoga OR yogi* OR asana* OR Iyengar OR ashtanga OR bikram OR hatha OR vinyasa OR tibetan OR kundalini OR (yoga AND (class* OR group* OR lesson* OR session* OR practice*)))<br>MeSH descriptor: [Yoga] explode all trees
#**3**	(online OR (web NEXT based) OR web OR (internet NEXT streamed) OR internet OR stream* OR digital* OR (home NEXT based) OR (home NEXT programme) OR home OR (at NEXT home) OR domiciliary OR videoconference OR (video NEXT based*) OR (app NEXT based*) OR application* OR iPad OR remote OR mobile OR (mobile NEXT app*) OR video* OR (device NEXT led) OR tele-yoga OR telehealth OR telemedicine OR e-health OR (provider NEXT led) OR supervised OR operator OR instructor* OR (instructor NEXT led*) OR guided OR live OR unsupervised OR (self NEXT guided) OR tutorial OR console OR (active NEXT video NEXT game*) OR (fitness NEXT game*) OR exergame OR gamification OR wii OR teacher* OR coach* OR trainer* OR tutor* OR guru* OR guide*)<br>MeSH descriptor: [Electronic Supplementary Materials] explode all trees<br>MeSH descriptor: [Telerehabilitation] explode all trees
