# Supplementary material for: Effects of Remotely Delivered Yoga Interventions on Quality of Life and Symptom Burden in Women with Breast Cancer: A Systematic Review and Meta-Analysis
Source: Healthcare (Basel). 2026 Sep 16;14(18):3040. doi: 10.3390/healthcare14183040 (PMC13606698; doi:10.3390/healthcare14183040)
Supplement: Supplementary file 1 [file healthcare-14-03040-s001.zip › Table S2.pdf]

Table S2. Outcome Measures Assessed in the Included Studies.

Outcome		RCTs n (%) Tot n 18	Quasi-experimental studies n (%) Tot n 8	Total publications n (%) Tot n 26
**Health-related Quality of life**		10 (55.6%)	3 (37.5%)	13 (50.0%)
**Feasibility/adherence**		8 (44.4%)	7 (87.5%)	15 (57.7%)
**Safety**		5 (27.8%)	4 (50%)	9 (34.6%)
**Psychological outcomes**	Anxiety	7 (38.9%)	3 (37.5%)	10 (38.5%)
	Depression	6 (33.3%)	2 (25.0%)	8 (30.8%)
	Stress	3 (16.7%)	1 (12.5%)	4 (15.4%)
	Sleep quality	4 (22.2%)	1 (12.5%)	5 (19.2%)
	Cognitive function	0 (0%)	3 (37.5%)	3 (11.5%)
	Mental well-being	1 (5.6%)	1 (12.5%)	2 (7.7%)
	Self-esteem/confidence	1 (5.6%)	0 (0%)	1 (3.8%)
	Fear of recurrence	0 (0%)	1 (12.5%)	1 (3.8%)
**Physical outcomes**	Cancer-related fatigue (CRF)	6 (33.3%)	3 (37.5%)	9 (34.6%)
	Lymphedema-related outcomes	4 (22.2%)	0 (0%)	4 (15.4%)
	Pain	1 (5.6%)	2 (25.0%)	3 (11.5%)
	Treatment-related side effects/toxicity	3 (16.7%)	0 (0%)	3 (11.5%)
	Mobility/physical activity	1 (5.6%)	2 (25.0%)	3 (11.5%)
	Waist/hip circumference and anthropometric measurements	1 (5.6%)	0 (0%)	1 (3.8%)
