# Supplementary material for: Effects of Remotely Delivered Yoga Interventions on Quality of Life and Symptom Burden in Women with Breast Cancer: A Systematic Review and Meta-Analysis
Source: Healthcare (Basel). 2026 Sep 16;14(18):3040. doi: 10.3390/healthcare14183040 (PMC13606698; doi:10.3390/healthcare14183040)
Supplement: Supplementary file 1 [file healthcare-14-03040-s001.zip › Table S3.pdf]

Table S3. JBI Critical Appraisal of the Included Randomized Controlled Trials (RCTs).

	Bias related to selection and allocation			Bias related to administration of intervention/exposure			Bias related to assessment, detection and measurement of the outcome			Bias related to participant retention	Statistical Conclusion Validity		
**Ref. n.**	**1. Was true randomization used for assignment of participants to treatment groups?**	**2. Was allocation to treatment groups concealed?**	**3. Were treatment groups similar at the baseline?**	**4. Were participants blind to treatment assignment?**	**5. Were those delivering the treatment blind to treatment assignment?**	**6. Were treatment groups treated identically other than the intervention of interest?**	**7. Were outcome assessors blind to treatment assignment?**	**8. Were outcomes measured in the same way for treatment groups?**	**9. Were outcomes measured in a reliable way**	**10. Was follow up complete and if not, were differences between groups in terms of their follow up adequately described and analysed?**	**11. Were participants analysed in the groups to which they were randomized?**	**12. Was appropriate statistical analysis used?**	**13. Was the trial design appropriate and any deviations from the standard RCT design (individual randomization, parallel groups) accounted for in the conduct and analysis of the trial?**
[51]	Y	Y	N	N	N	Y	N	Y	Y	Y	Y	Y	Y
[29]	Y	Y	Y	N	N	N	U	Y	Y	Y	Y	Y	Y
[48]	Y	Y	Y	N	N	Y	Y	Y	Y	Y	Y	Y	Y
[49]	Y	Y	Y	N	Y	Y	Y	Y	Y	Y	Y	Y	Y

[36]	Y	Y	Y	N	N	Y	N	Y	Y	Y	Y	Y	Y
[37]	Y	Y	Y	N	N	Y	N	Y	Y	Y	Y	Y	Y
[38]	Y	Y	Y	N	N	Y	Y	Y	Y	Y	Y	Y	Y
[39]	Y	Y	Y	N	N	Y	N	Y	Y	Y	Y	Y	Y
[40]	Y	Y	Y	N	N	Y	Y	Y	Y	Y	Y	Y	Y
[41]	Y	Y	Y	N	N	Y	Y	Y	Y	Y	Y	Y	Y
[42]	Y	Y	Y	N	N	Y	Y	Y	Y	Y	Y	Y	Y
[32]	Y	Y	Y	N	N	Y	N	Y	Y	Y	Y	Y	Y
[34]	N	Y	Y	N	N	Y	N	N	Y	Y	Y	Y	Y
[35]	Y	Y	Y	N	N	Y	N	Y	Y	Y	Y	Y	Y
[45]	Y	Y	Y	N	N	Y	N	Y	Y	Y	Y	Y	Y
[46]	Y	Y	Y	N	N	Y	Y	Y	Y	Y	Y	Y	Y
[50]	Y	Y	Y	N	N	Y	N	Y	Y	Y	Y	Y	Y
[47]	Y	Y	Y	N	N	Y	N	Y	Y	Y	Y	Y	Y
