# Supplementary material for: Effects of Remotely Delivered Yoga Interventions on Quality of Life and Symptom Burden in Women with Breast Cancer: A Systematic Review and Meta-Analysis
Source: Healthcare (Basel). 2026 Sep 16;14(18):3040. doi: 10.3390/healthcare14183040 (PMC13606698; doi:10.3390/healthcare14183040)
Supplement: Supplementary file 1 [file healthcare-14-03040-s001.zip › Table S4.pdf]

Table S4. JBI Critical Appraisal of the Included Quasi-Experimental Studies.

	**Temporal precedence**	**Selection and allocation**	**Confounding factors**	**Administration of intervention/exposure**	**Assessment, detection, and measurement of the outcome**			**Participant retention**	**Statistical Conclusion Validity**
	1. Is it clear in the study what is the "cause" and what is the "effect" (ie, there is no confusion about which variable comes first)?	2. Was there a control group?	3. Were participants included in any comparisons similar?	4. Were the participants included in any comparisons receiving similar treatment/care, other than the exposure or intervention of interest?	5. Were there multiple measurements of the outcome, both pre and post the intervention/exposure?	6. Were the outcomes of participants included in any comparisons measured in the same way?	7. Were outcomes measured in a reliable way?	8. Was follow-up complete and, if not, were differences between groups in terms of their follow-up adequately described and analyzed?	9. Was appropriate statistical analysis used?
**Ref. n.**									
[52]	U	N	Y	Y	Y	Y	N	Y	U
[43]	Y	N	Y	Y	Y	Y	N/A	Y	Y
[55]	Y	N	Y	Y	Y	Y	Y	Y	Y
[53]	Y	N	NA	NA	Y	NA	Y	Y	Y
[56]	Y	N	NA	NA	Y	NA	Y	Y	Y
[33]	Y	N	NA	NA	Y	NA	N	Y	N
[44]	Y	N	NA	NA	Y	NA	Y	Y	Y
[54]	Y	N	NA	Y	Y	NA	Y	Y	Y
