# Supplementary material for: Effects of Remotely Delivered Yoga Interventions on Quality of Life and Symptom Burden in Women with Breast Cancer: A Systematic Review and Meta-Analysis
Source: Healthcare (Basel). 2026 Sep 16;14(18):3040. doi: 10.3390/healthcare14183040 (PMC13606698; doi:10.3390/healthcare14183040)
Supplement: Supplementary file 1 [file healthcare-14-03040-s001.zip › Table S5.pdf]

Study	Included in qualitative synthesis	Included in primary meta-analysis	Reason for exclusion from primary meta-analysis
Littman et al., 2012	✓	✓	
Loudon et al., 2014	✓	✓	
Loudon et al., 2016	✓	✗	Same study population as Loudon 2014.
Prakash et al., 2017	✓	✗	Same study population as Prakash et al. (2020); duplicate cohort.
Prakash et al., 2020	✓	✓	
Harder et al., 2015	✓	✓	
Qi et al., 2026	✓	✓	
Đorđević et al., 2024	✓	✓	
Raghavendra et al., 2007	✓	✓	
Raghavendra Mohan Rao et al., 2017	✓	✗	Follow-up/secondary report of the same trial population published in 2007.
Lanctôt et al., 2015	✓	✗	Psychological outcome reported instead of HRQoL (not selected for the primary meta-analysis)
Anestin et al., 2022	✓	✗	Secondary analysis of the Lanctôt et al. trial; overlapping population.
Kovačič et al., 2011 (GHQ/RSCL, PSS)	✓	✗	Psychological outcome reported instead of HRQoL (not selected for the primary meta-analysis)
Kovačič et al., 2011 (Rosenberg Self-Esteem)	✓	✗	Psychological outcome reported instead of HRQoL (not selected for the primary meta-analysis)
Kovačič et al., 2012 (STAI)	✓	✗	Psychological outcome reported instead of HRQoL (not selected for the primary meta-analysis)
Stan et al., 2016	✓	✓	
Movafegh et al., 2025	✓	✓	
Ferrigno Guajardo et al., 2025	✓	✗	Insufficient quantitative data (means and SDs not reported; only regression coefficients/adjusted estimates available), preventing calculation f standardized mean differences.

Supplementary Table S5. Eligibility of Randomized Controlled Trials for Quantitative Synthesis. Eligibility of randomized controlled trials for quantitative synthesis, indicating inclusion in the primary meta-analysis, together with reasons for exclusion.
