# Supplementary material for: Effects of Remotely Delivered Yoga Interventions on Quality of Life and Symptom Burden in Women with Breast Cancer: A Systematic Review and Meta-Analysis
Source: Healthcare (Basel). 2026 Sep 16;14(18):3040. doi: 10.3390/healthcare14183040 (PMC13606698; doi:10.3390/healthcare14183040)
Supplement: Supplementary file 1 [file healthcare-14-03040-s001.zip › Table S6.pdf]

Study	HRQoL instrument	Data used	Yoga n	Mean	SD	Control n	Mean	SD	Score direction	Hedges' g
Littman et al.	FACT-G	Post-intervention	30	90.30	11.00	27	87.70	15.00	Higher = better	0.19
Loudon et al.	LYMQOL – Total QOL	Post-intervention	12	7.42	1.24	11	7.45	1.44	Higher = better	−0.02
Prakash et al.	EORTC QLQ-C30	Post-intervention	41	69.10	15.05	42	59.32	13.04	Higher = better	0.69
Harder et al.	FACT-B+4	Post-intervention	39	88.70	19.70	39	85.60	17.10	Higher = better	0.17
Qi et al.	FACT-B	Post-intervention	15	96.00	12.23	15	91.26	15.74	Higher = better	0.33
Đorđević et al.	EORTC QLQ-C30/BR23	Post-intervention	71	66.60	10.00	46	54.10	11.00	Higher = better	1.19
Stan et al.	FACT-B	Change from baseline	18	7.00	10.70	16	5.50	9.70	Higher = better	0.14
Raghavendra et al.	FLIC	Post-intervention	28	142.10	10.20	34	111.70	25.50	Higher = better	1.49
Movafegh et al.	LLIS	Post-intervention	48	44.65	7.32	48	49.27	8.61	Lower = better†	0.57†

Note. Values are reported as sample size (*n*), mean, and standard deviation (SD). Effect sizes were calculated as standardized mean differences using Hedges' *g*. Post-intervention values were used whenever available; for Stan et al., change-from-baseline values and corresponding SDs were used. Positive Hedges' *g* values consistently indicate an effect favouring the yoga intervention. †For LLIS, lower scores indicate better health status; therefore, the direction of the effect size was reversed to maintain a consistent interpretation across studies.
